# Morphological and functional abnormalities of ophthalmic artery in patients with ophthalmic vascular accidents

**DOI:** 10.3389/fmed.2024.1334455

**Published:** 2024-07-22

**Authors:** Benqi Zhao, Yicong Ji, Yangyang Xu, Wei Su, Shancheng Si

**Affiliations:** ^1^Department of Radiology, Beijing Tsinghua Changgung Hospital, School of Clinical Medicine, Tsinghua University, Beijing, China; ^2^Eye Center, Beijing Tsinghua Changgung Hospital, School of Clinical Medicine, Tsinghua University, Beijing, China; ^3^Department of Neurosurgery, Beijing Tsinghua Changgung Hospital, School of Clinical Medicine, Tsinghua University, Beijing, China

**Keywords:** ocular ischemic syndrome, ocular vascular accident, digital subtraction angiography, internal carotid artery, ophthalmic artery

## Abstract

**Background:**

By observing and comparing the morphological and functional differences of the ophthalmic artery (OA) in patients with ocular vascular accidents (OVAs) due to iatrogenic embolism or non-iatrogenic occlusion, we propose a classification system based on the characteristics of OA on invasive digital subtraction angiography (DSA).

**Methods:**

All patients undergoing ophthalmic arterial DSA within 1 week after the OVAs between January 2017 and December 2021 were enrolled and divided into different types, and the differences between iatrogenic embolism and non-iatrogenic occlusion categories were compared.

**Results:**

A total of 27 eyes of 27 patients were included in this study. Based on the results of carotid/intracranial arterial DSA, the morphological and functional abnormalities of OA with OVAs can be divided into five types. The proportion of males (7.14%), ocular ischemic syndrome (OIS) (0.00%) and neovascular glaucoma (NVG) (0.00%) in the iatrogenic embolism category was significantly lower than that (84.62, 61.54, and 69.23%, respectively) of the non-iatrogenic occlusion category (*p* < 0.001, *p* = 0.001, *p* < 0.001, respectively). However, the proportion of no light perception (NLP) (100%), anterior segment ischemia (ASI) (71.43%), and orbital involvement (ophthalmoplegia and ptosis, 42.86%) eventually occurring in the former was significantly greater than that in the latter (23.08, 0.00, 0.00%, respectively) (*p* < 0.001, *p* < 0.001, *p* = 0.010, respectively).

**Conclusion:**

Ocular vascular accidents can be divided into five types based on the characteristics of OA on DSA.

## Introduction

Ocular ischemic syndrome (OIS), which usually results from chronic ocular hypoperfusion, is clinically characterized by decreased visual acuity, ocular/orbital pain, elevated intraocular pressure (IOP), and even neovascular glaucoma (NVG), with rare occurrences of phthisis bulbi (PB) and ophthalmoplegia (1). However, ocular vascular accidents (OVAs) defined in this article are mostly caused by embolism, thrombosis, or atherosclerotic stenosis of the ophthalmic artery (OA) and its branches, all of which are acute and manifest as varying degrees of sudden vision loss with or without ophthalmoplegia, anterior segment ischemia (ASI), or subsequent PB.

With the increasing popularity of facial cosmetic filler injections, the clinical features and prognosis of OVAs caused by different filler materials (which we called iatrogenic embolism) are also becoming more diverse, which is very different from traditional atherosclerotic thrombosis and stenosis (which we called non-iatrogenic occlusion). The fillers used include autologous fat (2, 3), hyaluronic acid (HA) (2, 3), calcium hydroxyapatite (CaHA) (4), poly L-lactic acid (PLLA) (5) and corticosteroid suspension (CS) (6), etc. The size and physicochemical properties of their microspheres are different, and the type and scope of blood vessels embolized by fillers are different; therefore, the clinical manifestations and consequences are also different.

To the best of our knowledge, previous literature has rarely explored a classification system for OVAs based on OA observation, especially when multiple etiologies of iatrogenic embolism and non-iatrogenic occlusion are present. To compare the similarities and differences between different types of OVAs, and between iatrogenic embolism and non-iatrogenic occlusion categories, we reviewed all OVA patients who underwent carotid/intracranial arterial digital subtraction angiography (DSA) with intracranial arterial computed tomographic angiography (CTA). In addition, we systematically describe the clinical characteristics of different types of OVAs and discuss the anatomical basis and pathophysiological mechanisms underlying these features.

## Methods

### Subjects

After a comprehensive review, 27 eligible patients were included in the study. The study protocol was approved by the Ethics Committee and complied with the tenets of the Declaration of Helsinki. The Board waived the requirement for written consent because of the retrospective nature of the study. All analyzed data were anonymized and deidentified.

### Inclusion criteria

**Figure 1:**
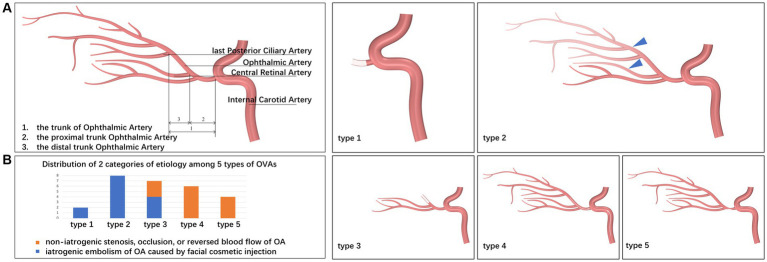
Schematic diagrams for segmentation of the OA and types of OVAs. **(A)** Schematic diagrams for segmentation of the OA. Based on the results of carotid/intracranial arterial DSA, the morphological and functional abnormalities of OA that caused OVAs can be divided into five types: OVA types 1 (no visualization of the proximal trunk OA), 2 (poor visualization of the distal trunk OA and most distal branches, blue triangles), 3 (no visualization of the distal trunk OA), 4 (reversed visualization of blood flow in the trunk OA), and 5 (visualization of the narrowed trunk OA). **(B)** Distribution of 2 categories of etiology among 5 types of OVAs. DSA, digital subtraction angiography; OA, ophthalmic artery; OVA(s), ocular vascular accident(s).

The inclusion criteria were as follows: (1) all patients experiencing OVAs between January 2017 and December 2021; and (2) all enrolled patients undergoing carotid/intracranial arterial DSA for OA assessment within 1 week after the OVAs; and (3) all enrolled patients with embolism, stenosis, or abnormal blood flow in the trunk OA. The trunk OA was defined as the segment from the origin of OA to the origin of the last posterior ciliary artery (PCA). The proximal trunk OA was defined as the segment from the origin of OA to the origin of the central retinal artery (CRA), not including the origin of the CRA. The distal trunk OA was defined as the segment from the origin of the CRA to the origin of the last PCA, including the origin of the CRA. Variety clinical characteristics of OVAs can be summarized into 5 types. Detailed segmentation of the OA and types of OVAs were depicted in the schematic diagram (Figure 1).

### Exclusion criteria

The exclusion criteria were as follows: (1) stenosis of ocular blood vessels caused by trauma, inflammation, or radiation therapy; (2) only branch lesions of the OA, without embolism or stenosis of the trunk OA, such as simple retinal artery occlusion (RAO) and simple anterior ischemic optic neuropathy (AION); (3) other ocular vascular diseases such as vitreous hemorrhage, retinal vein occlusion, ocular tumor, ocular vascular malformation, and proliferative diabetic retinopathy; (4) active syphilis, hepatitis B, or other infectious diseases; or (5) insufficient data, such as lack of imaging studies of the OA.

### Ocular/systemic examinations

All enrolled subjects were subjected to a series of ocular/systemic examinations, as follows: (1) best-corrected visual acuity (BCVA) assessment; (2) IOP obtained by non-contact tonometer (NCT); (3) slit-lamp microscopy; (4) fundus photography; (5) optical coherence tomography (OCT) (spectral-domain OCT, Heidelberg Engineering, Heidelberg, Germany); and (6) carotid/intracranial arterial DSA with CTA for confirmation of embolism, stenosis, or abnormal blood flow of the carotid/ocular artery. All enrolled patients underwent ocular examinations and OA assessment using carotid/intracranial arterial DSA at enrollment, and ocular examinations were performed again at the last follow-up 6 months after the onset of OVAs.

### Definitions of terms in this study

#### Ocular vascular accident

**Figure 2:**
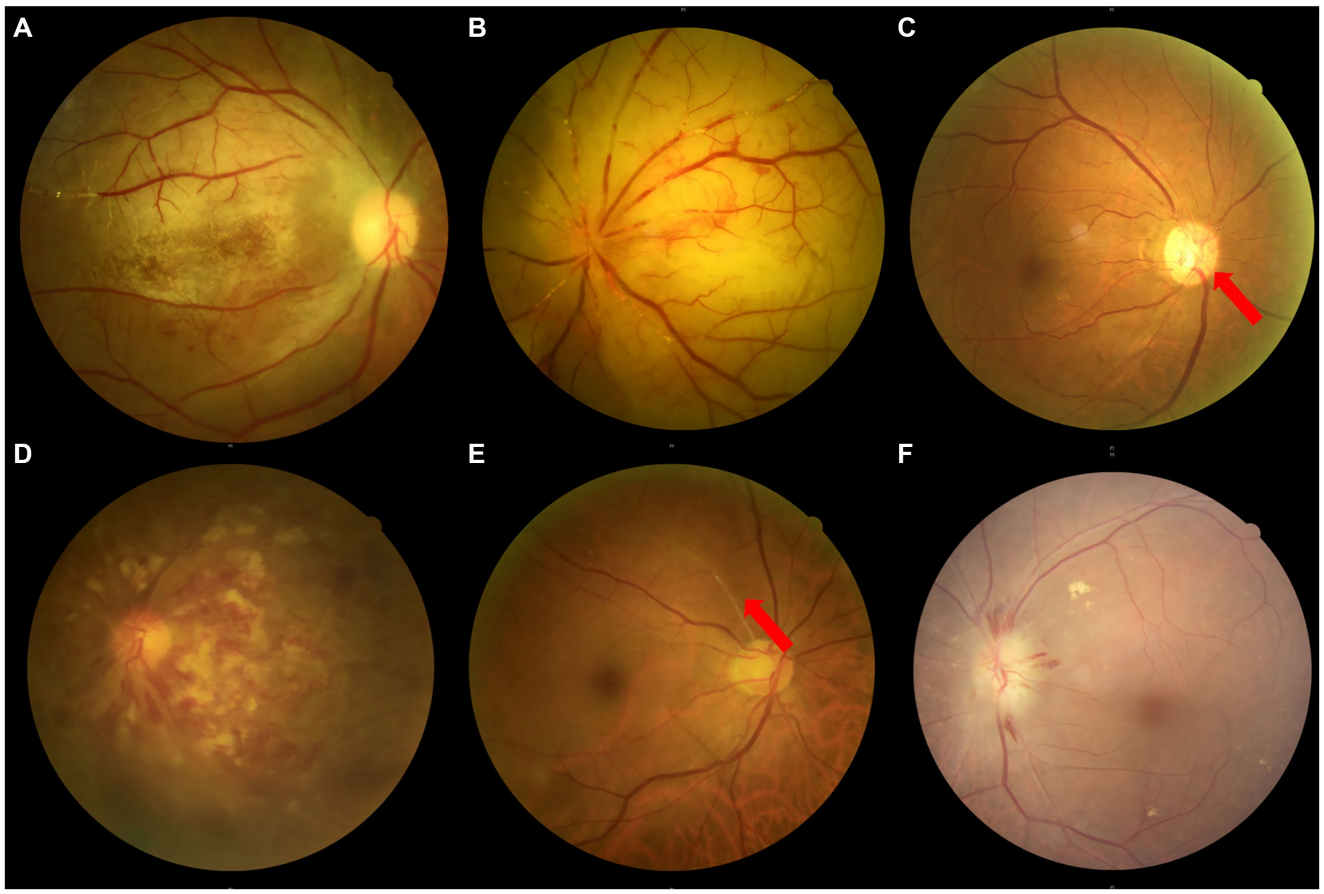
Fundus photographs of a variety of OVAs presenting with sudden blurred vision. **(A)** The OAO induced by autologous fat from Case No. 10, taken on the 18th day after the OVA onset. **(B)** The OAO induced by hyaluronic acid from Case No. 20, taken on the 7th day after the OVA onset. **(C)** The ocular ischemic syndrome presenting with OVA from Case No. 14, with a typical clinical feature of optic disc neovascularization (red arrow). **(D)** The central retinal vein occlusion presenting with OVA from Case No. 8. **(E)** The BRAO (red arrow) presenting with OVA from Case No. 24. **(F)** The AION presenting with OVA from Case No. 13. BRAO, branch retinal artery occlusion; OAO, ophthalmic artery occlusion, OVA(s), ocular vascular accident(s); AION, anterior ischemic optic neuropathy.

Sudden blurred vision due to embolism, stenosis, or abnormal blood flow of the trunk OA, rather than the diseases of eyeball itself. Specifically, OVAs include sudden embolization of the OA and its branches due to facial cosmetic fillers, suddenly acute attack of chronically progressive OIS or NVG due to stenosis or abnormal blood flow of the trunk OA, and sudden RAO or AION due to stenosis or abnormal blood flow of the trunk OA (Figure 2), but do not include vitreous hemorrhage, retinal detachment, and NVG attack et al.

#### Ophthalmic artery occlusion

**Figure 3:**
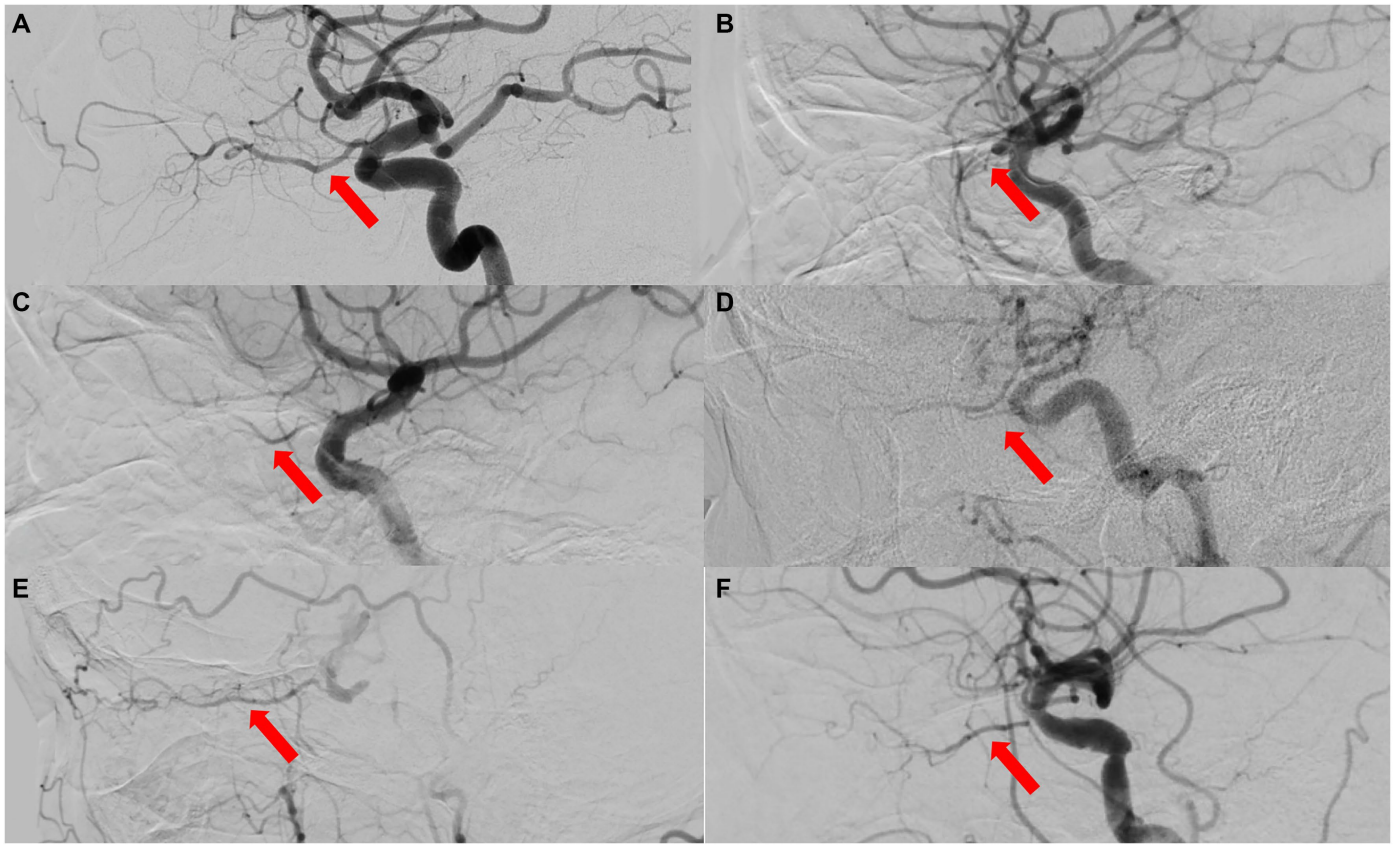
The ophthalmic artery features on DSA of 5 types of OVAs. **(A)** A normal control case presenting with good visualization of the trunk OA (red arrow) and distal branches of OA. **(B)** Type 1 OVA presenting with no visualization of the proximal trunk OA (red arrow) from Case No. 10. **(C)** Type 2 OVA presenting with poor visualization of the distal trunk OA (red arrow) and most distal branches from Case No. 9. **(D)** Type 3 OVA presenting with no visualization of the distal trunk OA (red arrow) from Case No. 25. **(E)** Type 4 OVA presenting with reversed visualization of blood flow in the trunk OA (red arrow) from Case No. 6. **(F)** Type 5 OVA presenting with visualization of the narrowed trunk OA (red arrow) from Case No. 13. DSA, digital subtraction angiography; OA, ophthalmic artery; OVA(s), ocular vascular accident(s).

**Figure 4:**
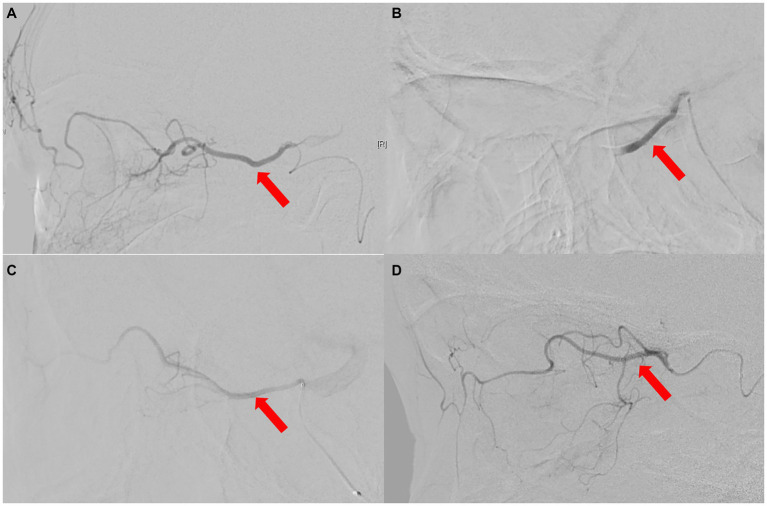
The ophthalmic artery features on selective ophthalmic DSA of 3 patients. **(A)** Normal control case presenting with perfect visualization of the trunk OA (red arrow) and distal branches of the OA. **(B)** Type 1 OVA with no visualization of the proximal trunk OA (red arrow) in Case No. 10. **(C,D)** Type 2 OVA presenting with poor visualization of the distal trunk OA (red arrow) and most distal branches from cases 9 and 1, respectively. DSA, digital subtraction angiography; OA, ophthalmic artery; OVA, ocular vascular accident.

Embolism or stenosis of the trunk OA, clinically characterized by sudden vision loss (almost no light perception, NLP), no visualization of the trunk OA on DSA/CTA, RAO or AION on fundus photography, choroidal hypoperfusion on indocyanine green angiography with or without ASI, ophthalmoplegia, etc. (Figures 1–4).

#### Phthisis bulbi

The pathological state with IOP of the affected eye usually less than 8 mmHg, shortened corneal diameter, thickened ocular coats, disorganized intraocular contents, and globe size 20% or more smaller than before injury (7).

#### Ocular ischemic syndrome

**Figure 5:**
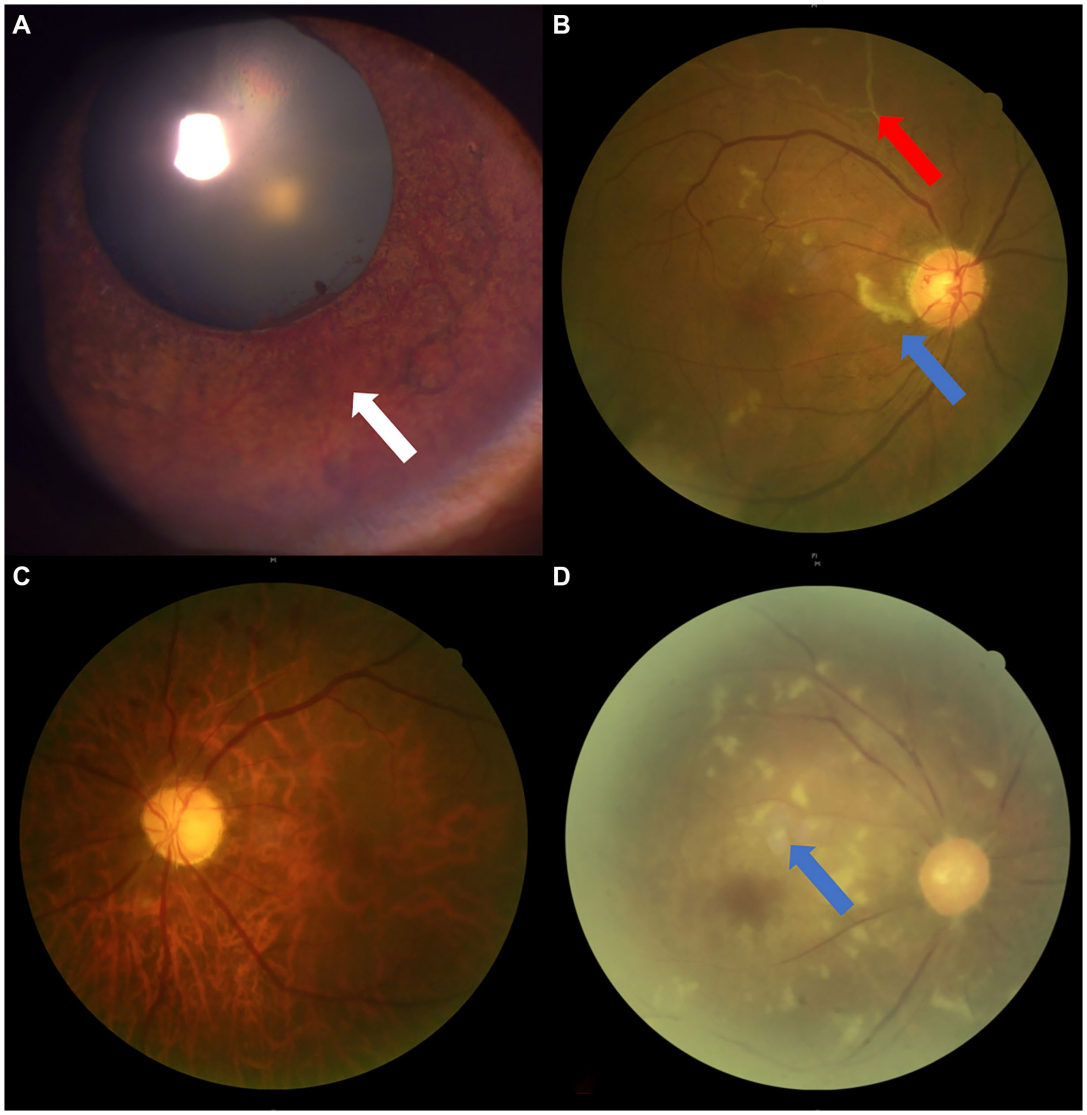
The ophthalmic imaging features of OIS. **(A,B)** Anterior/posterior segment photograph showing iris neovascularization (white arrow), RAO (red arrow), and cotton wool spots (blue arrow) in Case No. 6. **(C,D)** Fundus photographs showing RAO from Case No. 12 and pale optic disc/retina, retinal artery stenosis, and cotton wool spots (blue arrow) in Case No. 21. RAO, retinal artery occlusion; OIS, ocular ischemic syndrome.

Chronic progressive ocular ischemia due to ocular hypoperfusion, usually secondary to atherosclerotic carotid artery stenosis of more than 70%, clinically characterized by visual loss, amaurosis fugax, pain, anterior chamber cellular reaction, narrowing of retinal arteries, venous dilatation, peripheral retinal hemorrhages, cotton wool, patches vitreous hemorrhage, hyphema, optic disc and iris neovascularization (1) (Figure 5).

#### Anterior segment ischemia

The syndrome secondary to acute hypoperfusion of the anterior segment circulation caused by various reasons, with clinical characteristics of segmental or circumferential atrophy of the iris, pupil irregularity, uveitis, corneal edema, ciliary hyposecretion, hypotony, cataract, even PB (7).

### Statistical analyses

Based on the characteristics of OA on DSA, we first divided all OVAs into five types and described their demographic and multimodal imaging features. We then classified the five types into iatrogenic embolism and non-iatrogenic occlusion categories based on the pathophysiological mechanism, and compared the differences between the two categories. Also, based on different etiologies, all cases were divided into seven subcategories: autologous fat-induced ophthalmic artery occlusion (OAO), HA-induced incomplete OAO, HA-induced distal occlusion of OA, atherosclerotic occlusion of distal OA, ipsilateral internal carotid artery (internal carotid artery) complete occlusion (defined as 100% ICA stenosis), severe stenosis (defined as ≥70% and <100% ICA stenosis) of the ipsilateral ICA and varying degrees (defined as <70% ICA stenosis) of stenosis of the ipsilateral ICA or OA. Etiological distribution of five types of OVAs was described. Finally, we established a model for predicting reversed visualization of OA using imaging features of the ICA on minimally invasive CTA and calculated its sensitivity, specificity, and area under the receiver operating characteristic curve (AUC).

All data were processed and compared using the SPSS software for Windows (version 25.0, IBM Corp., Armonk, NY, United States). Continuous variables conforming to a normal distribution were compared using Student’s t-test; categorical variables were compared using Fisher’s exact test because of the small sample size (*p* < 0.05).

## Results

### Demographic and ophthalmic artery abnormalities on carotid/intracranial arterial DSA

A total of 27 eyes from 27 patients were included in this study, with females accounting for 55.60% (15 cases) and right eyes for 55.60% (15 eyes). Their average age was 49.7 ± 19.3 years (range, 21–84 years) and BCVA was mostly lower than 20/400, except in 4 patients (cases 12, 14, 24, 26), with NLP accounting for 63.00% (17 cases). Among them, three patients (11.11%) underwent selective ophthalmic DSA due to needing further treatments (Case Nos. 1, 9, 10, Figure 4).

Based on the results of carotid/intracranial arterial DSA, the morphological and functional abnormalities of OA that caused OVAs can be divided into five types: OVA type 1 (no visualization of the proximal trunk OA, 7.40%, 2 cases), OVA type 2 (poor visualization of the distal trunk OA and most distal branches, 29.60%, 8 cases), OVA type 3 (no visualization of the distal trunk OA, 25.90%, 7 cases), OVA type 4 (reversed visualization of blood flow in the trunk OA, 22.20%, 6 cases), and OVA type 5 (visualization of the narrowed trunk OA, 14.80%, 4 cases) (Figures 1, 3, 4). The detailed demographic and clinical characteristics (including sex, age, involved eye, BCVA, slit-lamp and fundus examinations, carotid/intracranial arterial DSA, and diagnosis) of all 27 cases at the initial visit are listed in Table 1.

**Table 1:** The demographic, clinical characteristics and OVA types of all 27 enrolled cases.

Case No.	Involved eye	BCVA	Slit-lamp/fundus examinations at first visit	Ophthalmic artery angiography	OVAs and subjective diagnosis	Types	Long-term complications affecting IOP
1	OS	NLP	Mydriasis, pale optic disc/retina, retinal intra-arterial emboli	Poor visualization of the distal trunk OA and most distal branches	Stroke, HA-induced CRAO, ASI	2	Hypotony
2	OD	NLP	Conjunctival congestion, cornea edema	No visualization of the distal trunk OA	HA-induced OAO, ophthalmoplegia, ptosis, ASI, PB	3	PB
3	OD	NLP	Conjunctival congestion, cornea edema	No visualization of the distal trunk OA	HA-induced OAO, ophthalmoplegia, ptosis, ASI, PB	3	PB
4	OD	NLP	Conjunctival congestion, cornea edema, hyphema	No visualization of the distal trunk OA	Stroke, HA-induced OAO, ophthalmoplegia, ptosis, ASI, PB	3	PB
5	OS	NLP	Mydriasis, pale optic disc/retina/choroid, retinal intra-arterial emboli	Poor visualization of the distal trunk OA and most distal branches	HA-induced OAO	2	—
6	OD	20/400	Mydriasis, iris and optic disc neovascularization, retinal artery occlusion and cotton wool spots	Reversed visualization of blood flow in the trunk OA	Stroke, NVG secondary to OIS, ipsilateral ICAO	4	NVG
7	OD	NLP	Mydriasis, pale optic disc/retina/choroid, retinal intra-arterial emboli, punctate hemorrhage on the retinal surface	No visualization of the proximal trunk OA	Autologous fat induced OAO	1	—
8	OD	LP	Mydriasis, iris neovascularization, extensive retinal artery occlusion	No visualization of the distal trunk OA	NVG secondary to CRVO, ipsilateral OAO, 20% stenosis of ipsilateral ICA, contralateral ICAO	3	NVG
9	OS	Delayed-NLP	Mydriasis, pale and swollen optic disc, several triangular choroidal non-perfused areas	Poor visualization of the distal trunk OA and most distal branches	HA-induced AION, HA-induced ChAO	2	—
10	OD	NLP	Mydriasis, pale optic disc/retina/choroid, retinal intra-arterial emboli	No visualization of the proximal trunk OA	Stroke, autologous fat induced OAO, ophthalmoplegia, ptosis, ASI	1	—
11	OD	HM	Mydriasis, iris and optic disc neovascularization, retinal artery stenosis and cotton wool spots	Reversed visualization of blood flow in the trunk OA	NVG secondary to OIS, ipsilateral ICAO	4	NVG
12	OS	20/200	Mydriasis, iris neovascularization, retinal artery occlusion and patchy hemorrhage	Reversed visualization of blood flow in the trunk OA	NVG secondary to OIS, ipsilateral ICAO	4	NVG
13	OS	NLP	Mydriasis, swollen and hemorrhagic optic disc	Visualization of the narrowed trunk OA	AION, stenosis of ipsilateral OA	5	—
14	OD	20/80	Iris and optic disc neovascularization, retinal artery stenosis	No visualization of the distal trunk OA	Stroke, NVG secondary to OIS, ipsilateral OAO, 50% stenosis of ipsilateral ICA	3	NVG
15	OS	FC	Mydriasis, hyphema, iris and optic disc neovascularization, retinal artery stenosis	Reversed visualization of blood flow in the trunk OA	Stroke, NVG secondary to OIS, ipsilateral ICAO	4	NVG
16	OD	NLP	Mydriasis, pale optic disc/retina/choroid, retinal intra-arterial emboli	Poor visualization of the distal trunk OA and most distal branches	HA-induced OAO, ASI	2	CHS
17	OS	NLP	Mydriasis, pale optic disc/retina/choroid, retinal intra-arterial emboli	Poor visualization of the distal trunk OA and most distal branches	HA-induced OAO, ASI, ophthalmoplegia, ptosis	2	—
18	OD	NLP	Conjunctival congestion, cornea edema, uveitis, mydriasis	No visualization of the distal trunk OA	HA-induced OAO, ophthalmoplegia, ptosis, ASI, PB	3	PB
19	OS	NLP	Mydriasis, pale optic disc/retina/choroid	Poor visualization of the distal trunk OA and most distal branches	HA-induced OAO	2	—
20	OS	NLP	Uveitis, pigmentation on the lens surface, mydriasis, pale optic disc/retina/choroid, retinal intra-arterial emboli	Poor visualization of the distal trunk OA and most distal branches	HA-induced OAO, ASI	2	Hypotony
21	OD	NLP	Mydriasis, iris neovascularization, pale optic disc/retina, retinal artery stenosis, and cotton wool spots	Reversed visualization of blood flow in the trunk OA	Stroke, NVG secondary to OIS, 70% stenosis of ipsilateral ICA, contralateral ICAO	4	NVG
22	OS	NLP	Uveitis, iris atrophy of 1 quadrant, pigmentation on the lens surface, mydriasis, pale optic disc/retina/choroid, retinal intra-arterial emboli	Poor visualization of the distal trunk OA and most distal branches	HA-induced OAO, ASI	2	Hypotony
23	OD	NLP	Mydriasis, hyphema, iris neovascularization, vitreous hemorrhage	Reversed visualization of blood flow in the trunk OA	NVG secondary to OIS, 80% stenosis of ipsilateral ICA	4	NVG
24	OD	20/40	Superior temporal BRAO with pallor edema in the corresponding blood supply retina	Visualization of the narrowed trunk OA	Stroke, BRAO, stenosis of ipsilateral OA, 50% stenosis of ipsilateral ICA	5	—
25	OS	LP	Mydriasis, iris neovascularization, optic atrophy, retinal arterial occlusion	No visualization of the distal trunk OA	Stroke, NVG secondary to PDR, OAO, 50% stenosis of ipsilateral ICA	3	NVG
26	OD	20/40	Optic disc neovascularization, vitreous hemorrhage	Visualization of the narrowed trunk OA	VH secondary to OIS, stenosis of ipsilateral OA; 60% stenosis of ipsilateral ICA	5	—
27	OS	FC	Superficial panretinal hemorrhage, retinal artery stenosis, and cotton wool spots	Visualization of the narrowed trunk OA	CRVO, CRAO, stenosis of ipsilateral OA	5	—

AION, anterior ischemic optic neuropathy; ASI, anterior segment ischemia; BCVA, best corrected visual acuity; BRAO, branch retinal artery occlusion; CRVO, central retinal vein occlusion; ChAO, choroidal artery occlusion; CHS, ciliary hyposecretion; FC, finger counting; HA, hyaluronic acid; HM, hand motion; ICA, internal carotid artery; ICAO, internal carotid artery occlusion; IOP, intraocular pressure; LP, light perception; NLP, no light perception; NVG, neovascular glaucoma; OA, ophthalmic artery; OAO, ophthalmic artery occlusion; OIS, ocular ischemic syndrome; OVA, ocular vascular accident; PB, phthisis bulbi; PDR, proliferative diabetic retinopathy; VH, vitreous hemorrhage.

### Multimodal imaging features and etiological analysis of five types of OVAs

**Figure 6:**
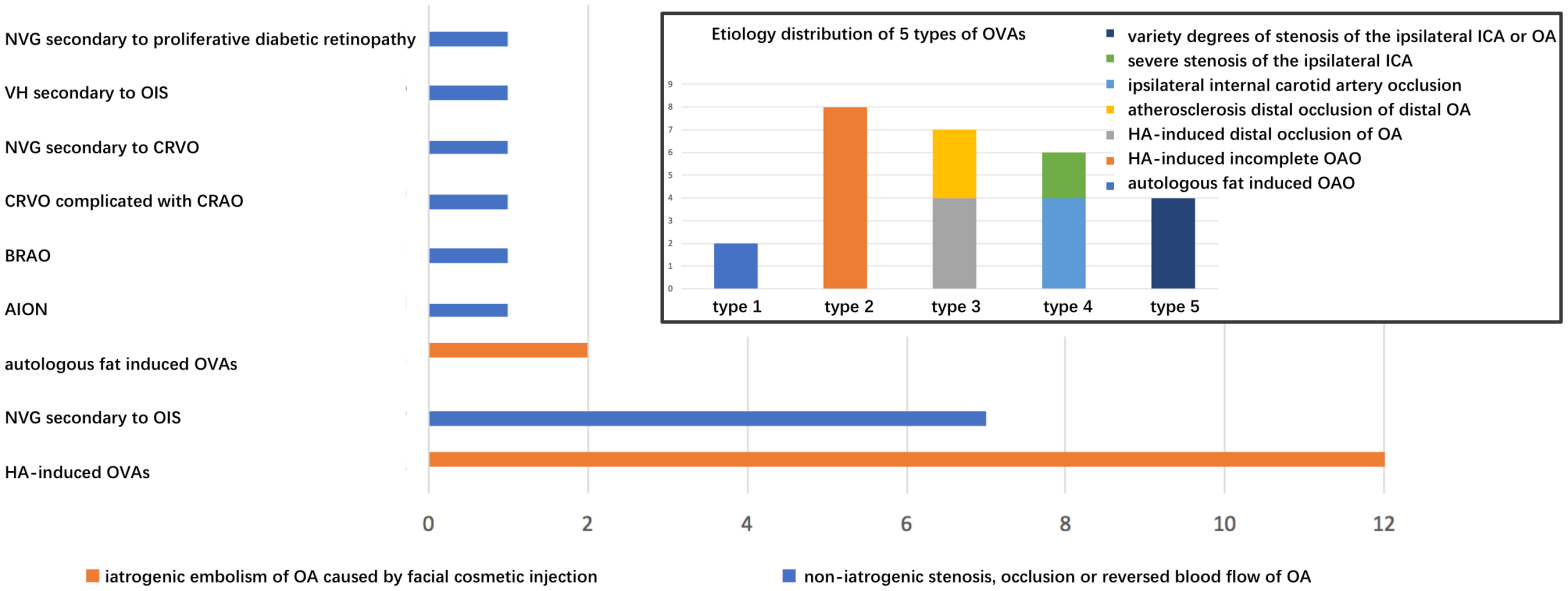
Distribution of related ophthalmic diseases in 2 categories of etiology. The inset showing etiological distribution of 5 types of OVAs. OVAs, ocular vascular accidents.

Among the 27 cases described in this article, OVA type 1 resulted from autologous fat-induced OAO (7.40%, 2 cases, including cases 7 and 10) (Figure 2A), and OVA type 2 was caused by HA-induced incomplete OAO (29.60%, 8 cases) (Figure 2B). OVA type 3 mainly resulted from HA-induced distal occlusion of the OA (14.80%, 4 cases, including cases 2, 3, 4 and 18) and atherosclerotic occlusion of the distal OA (11.10%, 3 cases, including cases 8, 14 and 25) (Figures 2C,D). OVA type 4 was mostly caused by ipsilateral internal carotid artery occlusion (ICAO) (14.80%, 4 cases, including cases 6, 11, 12 and 15), with the exception of 2 cases caused by severe stenosis of the ipsilateral ICA (7.40%, 2 cases, including cases 21 and 23). In addition, OVA type 5 resulted from varying degrees of stenosis of the ipsilateral ICA or OA (14.80%, four cases, including cases 13, 24, 26 and 27) (Figures 2E,F). The main multimodal imaging features and etiology of the five OVAs are listed in Table 2. The etiological distribution of 5 types of OVAs was summarized in Figure 6 (inset).

**Table 2:** Main multimodal imaging features and etiology of five types of OVAs.

OVA types	Carotid/intracranial arterial DSA	Multimodal imaging features			Etiology
		Slit-lamp photo	Color fundus photo	Imaging features of OA on CTA	
1	No visualization of the proximal trunk OA	Mydriasis	Pale optic disc/retina/choroid, retinal intra-arterial emboli, punctate hemorrhage on the retinal surface	No visualization of the proximal trunk OA	Autologous fat induced OAO
2	Poor visualization of the distal trunk OA and most distal branches	Uveitis, iris atrophy, pigmentation on the lens surface, mydriasis	Pale optic disc/retina/choroid, retinal intra-arterial emboli, triangular choroidal non-perfused areas	Poor or no visualization of the distal trunk OA	HA-induced incomplete OAO
3	No visualization of the distal trunk OA	Conjunctival congestion, cornea edema, hyphema, uveitis, iris neovascularization	Optic atrophy, extensive retinal artery occlusion	No visualization of the distal trunk OA	HA-induced or atherosclerotic occlusion of distal OA
4	Reversed visualization of blood flow in the trunk OA	Mydriasis, hyphema, iris neovascularization	Optic disc neovascularization, retinal artery occlusion and cotton wool spots, pale optic disc/retina, vitreous hemorrhage	Poor visualization of the distal trunk OA	Ipsilateral internal carotid artery occlusion or severe stenosis^a^
5	Visualization of the narrowed trunk OA	Mydriasis	Swollen and hemorrhagic optic disc, RAO with pallor edema in the corresponding blood supply retina, Optic disc neovascularization, vitreous hemorrhage	Poor or no visualization of the distal trunk OA	Variety degrees of stenosis of the ipsilateral ICA or OA

CTA, computed tomographic angiography; DSA, digital subtraction angiography; HA, hyaluronic acid; ICA, internal carotid artery; OA, ophthalmic artery; OAO, ophthalmic artery occlusion; OVA, ocular vascular accident; RAO, retinal artery occlusion.

a Severe stenosis of ipsilateral ICA indicates 70–99% stenosis of ipsilateral ICA.

### Comparison between two categories of abnormalities of OA

**Figure 7:**
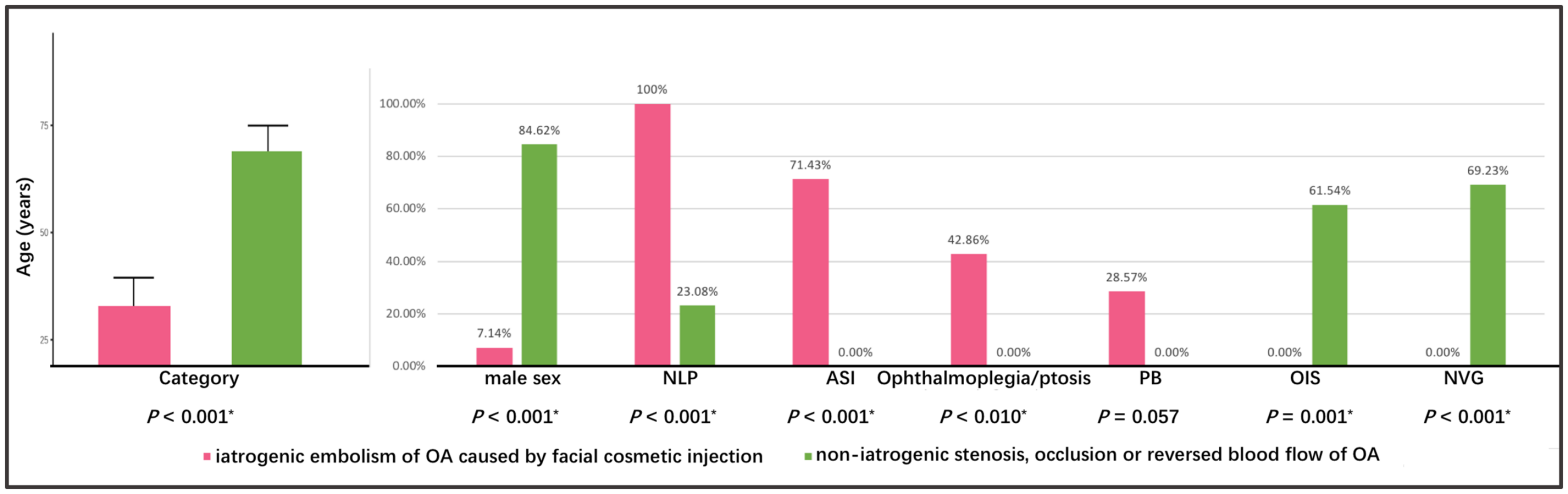
Comparison of demographic and clinical features between two categories of abnormalities of OA. The asterisk (*) indicates *p* < 0.05. OA, ophthalmic artery.

Based on the pathophysiological mechanism of OVAs, the etiology of the five types of abnormalities of OA can be classified into iatrogenic embolism and non-iatrogenic occlusion categories (including stenosis, occlusion, or reversed blood flow in OA). The detailed distribution of two categories in different OVA types and diseases was depicted in Figures 1, 6. It showed that age (32.57 ± 6.89 years), male percentage (7.14%), proportion undergoing OIS (0.00%) and NVG (0.00%) in iatrogenic embolism category was significantly lower than that (68.08 ± 6.86 years, 84.62, 61.54, 69.23%, respectively) in non-iatrogenic occlusion category (*p* < 0.001, *p* < 0.001, *p* = 0.001, *p* < 0.001, respectively). However, the proportions of NLP (100%), ASI (71.43%), and orbital involvement (ophthalmoplegia and ptosis, 42.86%) eventually occurring in the former were significantly greater than those (23.08, 0.00, 0.00%, respectively) in the latter (*p* < 0.001, *p* < 0.001, *p* = 0.010, respectively). Note that four (28.57%) and zero (0.00%) cases of PB occurred in the two categories, respectively, but there was no statistical difference between them (*p* = 0.057). A comparison of the demographic and clinical features of the two categories of OA abnormalities is listed in Table 3 and was depicted in Figure 7.

**Table 3:** Comparison of demographic and disease features between two categories of abnormalities of OA.

Variables	Overall (*n* = 27)	Categories of Abnormalities of OA		*p*-value
		Iatrogenic embolism (*n* = 14)	Non-iatrogenic occlusion (*n* = 13)	
**Demographic**				
Male sex	12 (44.44)	1 (7.14)	11 (84.62)	<0.001**
Age (yrs)	49.67 ± 19.30	32.57 ± 6.89	68.08 ± 6.86	<0.001**
Right eye	15 (55.56)	7 (50.00)	8 (61.54)	0.704
**Clinical features**				
NLP	17 (62.96)	14 (100.00)	3 (23.08)	<0.001**
ASI	10 (37.04)	10 (71.43)	0 (0.00)	<0.001**
**Ophthalmoplegia and ptosis**	6 (22.22)	6 (42.86)	0 (0.00)	0.016*
PB	4 (14.81)	4 (28.57)	0 (0.00)	0.098
OIS	8 (29.63)	0 (0.00)	8 (61.54)	0.001*
NVG	9 (33.33)	0 (0.00)	9 (69.23)	<0.001**
Stroke	9 (33.33)	3 (21.43)	6 (61.54)	0.236
**Imaging features**				
Ipsilateral ICAO or severe stenosis of ipsilateral ICA^a^ on CTA	6 (22.22)	0 (0.00)	6 (46.15)	0.027*
Reversed blood flow of OA on DSA	6 (22.22)	0 (0.00)	6 (46.15)	0.027*
**OVA types**				
Type 1 OVA	2 (7.41)	2 (14.29)	0 (0.00)	0.481
Type 2 OVA	8 (29.63)	8 (57.14)	0 (0.00)	0.002*
Type 3 OVA	7 (25.93)	4 (28.57)	3 (23.08)	1.000
Type 4 OVA	6 (22.22)	0 (0.00)	6 (46.15)	0.027*
Type 5 OVA	4 (14.81)	0 (0.00)	4 (30.77)	0.041*

ASI, anterior segment ischemia; BCVA, best corrected visual acuity; CTA, computed tomographic angiography; DSA, digital subtraction angiography; ICA, internal carotid artery; ICAO, internal carotid artery occlusion; NLP, no light perception; NVG, neovascular glaucoma; OA, ophthalmic artery; OIS, ocular ischemic syndrome; OVA, ocular vascular accident; PB, phthisis bulbi. * = *p* < 0.05; ** = *p* < 0.001. Data were presented as mean ± standard deviation or No. (%). *p* < 0.05 was considered to be statistically significant.

a Severe stenosis of ipsilateral ICA indicates 70–99% stenosis of ipsilateral ICA.

### A useful predictive model for type 4 OVA by common clinical and imaging features

**Figure 8:**
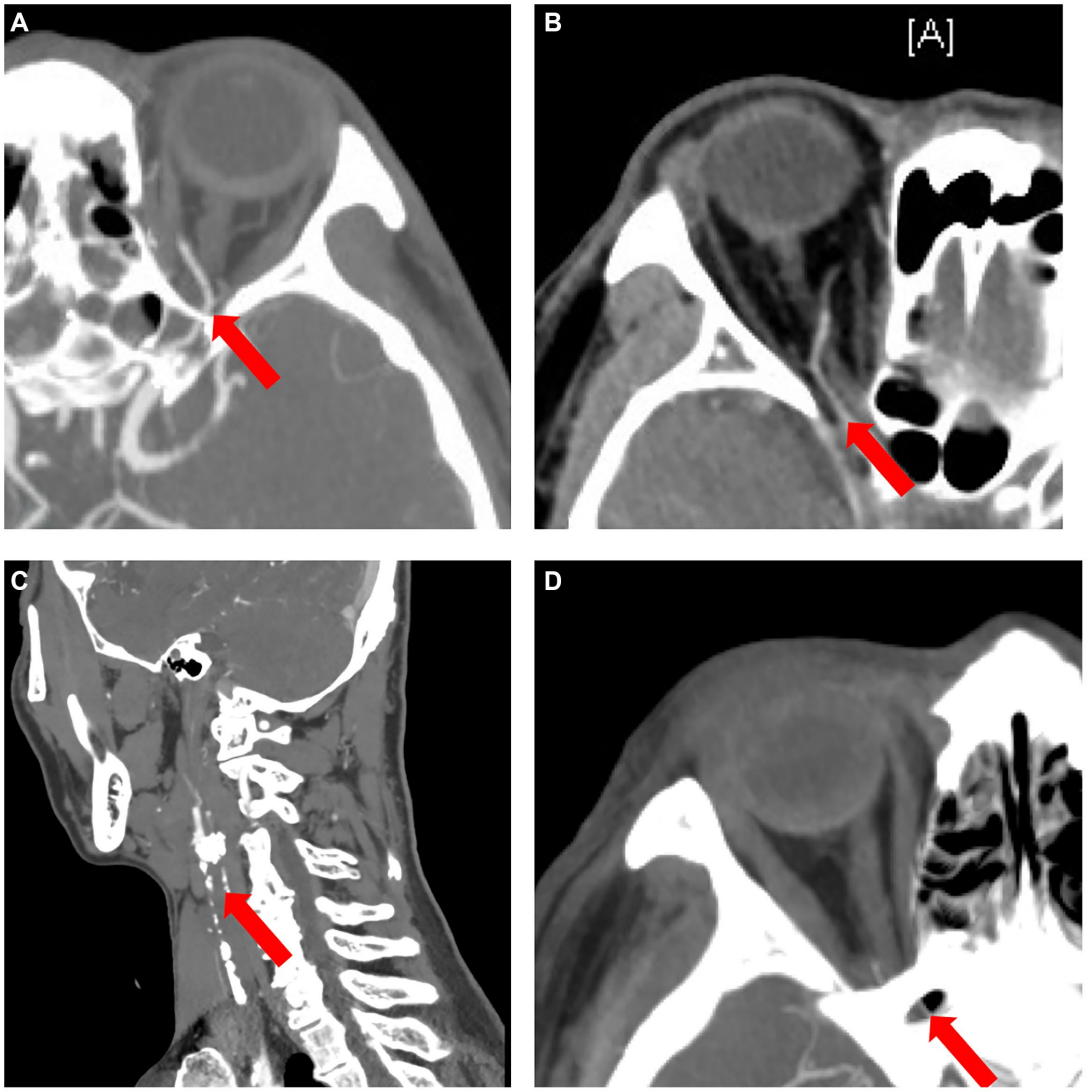
The ophthalmic artery features on CTA. **(A)** Normal control case presenting with good visualization of the trunk OA (red arrow) of the OA. **(B)** Type 4 OVA presenting with reversed visualization of blood flow in the trunk OA (red arrow) on DSA from Case No. 6, also showing a narrowed and stiff OA similar to the type 5 OVA on CTA. **(C)** Another type 4 OVA showing complete ICAO (red arrow) on CTA in Case No. 11. **(D)** Type 3 OVA presenting with no visualization of the distal trunk OA (red arrow) on DSA, also showing no visualization of the OA on CTA in Case No. 14. CTA, computed tomographic angiography; DSA, digital subtraction angiography; ICAO, internal carotid artery occlusion; OA, ophthalmic artery; OVA, ocular vascular accident.

Of the 5 types, type 4 OVA presenting with reversed blood flow of OA on DSA (6 cases) was the most dangerous, with the probability of OIS, NVG and stroke being 100% (6 cases), 100% (6 cases), and 50% (3 cases), respectively. Of the 27 enrolled cases, OIS, NVG, ipsilateral ICAO, and severe (≥ 70%) stenosis of the ipsilateral ICA on CTA were individual clinical features that were potentially minimally invasive predictors for type 4 OVA. AUCs for predicting type 4 OVA were 0.95, 0.93, and 1.00, respectively. The sensitivity, specificity, and AUC for the seven scenarios (A, B, C, D, E, F, and G) are listed in Table 4. The ophthalmic artery features on CTA are depicted in Figure 8.

**Table 4:** Sensitivity, specificity and AUC for predicting type 4 OVA by each scenario in this study.

Scenarios	Features	Sensitivity	Specificity	1-specificity	AUC	*p*-value
A	OIS	6/6 (1.00)	19/21 (0.90)	0.10	0.95	0.001*
B	NVG	6/6 (1.00)	18/21 (0.86)	0.14	0.93	0.002*
C	Ipsilateral ICAO or ≥80% stenosis of ipsilateral ICA on CTA	5/6 (0.83)	21/21 (1.00)	0.00	0.92	0.002*
D	Ipsilateral ICAO or ≥70% stenosis of ipsilateral ICA on CTA	6/6 (1.00)	21/21 (1.00)	0.00	1.00	<0.001**
E	Ipsilateral ICAO or ≥30% stenosis of ipsilateral ICA on CTA	6/6 (1.00)	17/21 (0.81)	0.19	0.90	0.003*
F	A and ≥30% stenosis of ipsilateral ICA on CTA	6/6 (1.00)	19/21 (0.90)	0.10	0.95	0.001*
G	B and ≥30% stenosis of ipsilateral ICA on CTA	6/6 (1.00)	19/21 (0.90)	0.10	0.95	0.001*

AUC, area under the receiver operating characteristic curve; CTA, computed tomographic angiography; ICA, internal carotid artery; ICAO, internal carotid artery occlusion; NVG, neovascular glaucoma; OIS, ocular ischemic syndrome. * = *p* < 0.05; ** = *p* < 0.001.

The best individual CTA feature for predicting type 4 OVA was scenario D [ipsilateral ICAO or severe (≥ 70%) stenosis of the ipsilateral ICA ≥70%]. The sensitivity, specificity, and AUC were 100, 100%, and 1.00, respectively (*p* < 0.001). When ≥30% stenosis of the ipsilateral ICA occurred, the best combination of features was F and G. That is, their predictive performance was the same when ≥30% stenosis of the ipsilateral ICA was combined with OIS or NVG, with a sensitivity, specificity, and AUC of 100, 90%, and 0.95, respectively (*p* = 0.001).

## Discussion

In our study, all type 1 OVAs presented with no visualization of the proximal trunk OA resulting from autologous fat-induced OAO. However, all type 2 OVAs and most type 3 OVAs are caused by HA-induced OAO. We speculate that this was related to the size of the filler particles. The largest diameter is autologous fat (20–1,400 μm) (3), followed by HA (350–900 μm) (3) and other filler materials [including CaHA (8), PLLA (5), CS, etc.] (Table 5). The result is that autologous fat is more likely to embolize proximal OA with a larger diameter of 1,250 ± 230 μm (10), leading to NLP (type 1 OVA), while HA tends to embolize distal OA, anterior segment circulation, as well as CRA with a larger diameter of 120–170 μm (9, 11), resulting in NLP, ophthalmoplegia, ASI, and PB (type 2/3 OVA); for the same reason, other fillers with smaller diameters can only embolize the blood vessels with smaller diameters than the CRA [such as the blood vessels of conjunctiva and extraocular muscles (5)], causing varying degrees of visual impairment, ophthalmoplegia, and ASI, rarely causing severe complications such as stroke and PB.

**Table 5:** Diameter size and clinical characteristics of different filler materials.

Materials	Diameter of microspheres (um)	Main clinical characteristics					
		NLP	Stroke	Ophthalmoplegia	ASI	PB	OVA types
Autologous fat	20–1,400	80.9% (2)	40.4–50% (2, 3)	40.4–66.7% (2, 3)	50% (3)	10% (3)	1
HA	350–900	39.1% (2)	8.7–20% (2, 3)	41.7–78.6% (2, 3)	50% (3)	20% (3)	2
CaHA	25–45 (9)	40%	10%	60%	60%	Rarely	2
PLLA	40–63 (5)	Rarely	Rarely	Sometimes	Sometimes	Rarely	2
CS^a^	<15	Rarely	Rarely	Sometimes	Rarely	Rarely	2

ASI, anterior segment ischemia; CaHA, calcium hydroxylapatite; CHS, ciliary hyposecretion; CS, corticosteroid suspension; HA, hyaluronic acid; NLP, no light perception; OVA, ocular vascular accident; PB, phthisis bulbi; PLLA, poly-L-lactic acid.

a The data comes from the 2020 edition of the Pharmacopoeia of the People’s Republic of China.

All type 4 OVAs are characterized by reversed blood flow of the OA, mostly caused by ICAO, and partially caused by severe (≥ 70%) stenosis of the ICA. As the stenosis of the ipsilateral ICA progressively worsens, the possibility of reversal of blood flow in the OA gradually increases, which is consistent with literature reports (12). The reversal of OA occurred in 6 of 7 patients presenting with OIS (85.71%), similar to that (75.00, 87.50, 66.70%, respectively) reported in the previous three literatures (12–14). It should be noted that all 6 cases with reversed blood flow of OA were complicated with stroke, suggesting that type 4 OVA means severe intracranial hypoperfusion, and the body has to open the external carotid artery-ophthalmic artery route to relieve intracranial hypoperfusion when the ICA is severely stenotic. According to reports by Kawaguchi et al. (12), among 8 cases with OIS, 7 cases had reversed blood flow in the ipsilateral OA, and 7 had ≥90% stenosis of the ipsilateral ICA.

It is important to note that ASI was observed in 71.43% of iatrogenic embolism and the proportion of NVG was much lower in the same group. Most of the iatrogenic embolism cases were caused by HA, and the small HA particles (usually 350–900 μm) (3) can completely block the anterior segment circulation of the eyeball and the greater arterial circle of iris. Due to the acute ASI event, the eyeball cannot form compensatory blood supply through collateral circulation, which can be confirmed on DSA, resulting in extensive ciliary process ischemic necrosis and subsequent hypotony or PB. After 2 years of follow-up, these acute cases of severe ASI rarely developed NVG (7). Because severe ciliary process necrosis resulted in severe ciliary process hyposecretion, IOP did not rise even if new vessels completely blocked the anterior chamber angle. Actually, all enrolled patients were followed for more than 6 months, and we did not find NVG occurring in the iatrogenic embolization category. In addition, although some patients with OIS and CRVO had already developed NVG at enrollment, our follow-up period was long enough (at least 6 months) to draw relatively objective conclusions. There were more female patients in the iatrogenic embolization category, which is similar to previous studies (3, 7). The difference in gender distribution reflected the fact that the female population received more facial cosmetic filler injections and the difference was not a bias.

It is well known that DSA remains the gold standard for diagnosing structural and functional abnormalities of OA to date. However, it is invasive and difficult to use as an outpatient screening tool for OA. In addition to DSA, minimally invasive CTA is the best way to evaluate the entire ICA and OA, followed by Doppler ultrasonography (15–17). Fortunately, we found that minimally invasive CTA was a good predictor for DSA outcomes in assessing the entire ICA and OA. Päivänsalo et al. (18) found that the orbital blood flow velocity decreases significantly when the stenosis of the ipsilateral ICA exceeds 80%. However, it has also been reported in the literature (13) that blood flow reversal of OA occurred in one patient, even if the stenosis of the ipsilateral ICA was approximately 70%. Therefore, we defined severe stenosis as ≥70% ICA stenosis.

This study has some limitations that cannot be ignored. First, this was a retrospective study on OVA patients. Although we collected all cases of OVAs within 5 years, only 27 cases were eligible for inclusion due to the invasive nature of DSA. Because of the relatively small sample, we cannot completely rule out the possibility of type 6 OVA, based on morphological and functional abnormalities of OA on DSA. Second, our study lacked functional observations, such as blood flow velocity of OA and ICA. Third, because DSA is an invasive test, the vast majority of the enrolled patients did not undergo re-examination of DSA at follow-up, which makes it impossible to assess whether the initial classification has changed at subsequent follow-up visits. In addition, AION, RVO and RAO are not directly related to OA, but may be complicated by OA abnormalities. In fact, in this study, we included a variety of diseases with different etiologies, which may increase selection bias. However, the classification system we proposed is mainly based on morphological differences in OA abnormalities rather than etiological differences. So, it’s not entirely unreasonable to classify them in the same type. We will continue to test the rationality of the proposed classification system in a future work.

To sum up, structural and functional abnormalities of the OAs on DSA can lead to 5 types of OVAs with different clinical manifestations such as OAO, ASI, OIS, and NVG, etc.; every type of OVA in turn lead to corresponding complications. Despite some shortcomings, we still believe that the classification of OVAs proposed in our article will help ophthalmologists to better understand the close relationship between OA and OVA. Nevertheless, further exploration and improvements are required.

## Data availability statement

The original contributions presented in the study are included in the article/supplementary material, further inquiries can be directed to the corresponding author.

## Ethics statement

The studies involving humans were approved by Beijing Tsinghua Changgung Hospital Ethics Committee (Approval No. 23300-6-01). The studies were conducted in accordance with the local legislation and institutional requirements. The ethics committee/institutional review board waived the requirement of written informed consent for participation from the participants or the participants’ legal guardians/next of kin because of the retrospective nature of the study. All analyzed data were anonymized and de-identified.

## Author contributions

BZ: Resources, Writing – original draft. YJ: Data curation, Writing – review & editing. YX: Data curation, Writing – review & editing. WS: Investigation, Writing – review & editing. SS: Project administration, Writing – review & editing, Writing – original draft.

## Glossary

AION: Anterior ischemic optic neuropathy
ASI: Anterior segment ischemia
AUC: Area under the receiver operating characteristic curve
BCVA: Best corrected visual acuity
BRAO: Branch retinal artery occlusion
CaHA: Calcium hydroxyapatite
CRA: Central retinal artery
CRVO: Central retinal vein occlusion
ChAO: Choroidal artery occlusion
CHS: Ciliary hyposecretion
CS: Corticosteroid suspension
CTA: Computed tomographic angiography
DSA: Digital subtraction angiography
FC: Finger counting
HA: Hyaluronic acid
HM: Hand motion
ICA: Internal carotid artery
ICAO: Internal carotid artery occlusion
IOP: Intraocular pressure
LP: Light perception
NCT: Non-contact tonometer
NLP: No light perception
NVG: Neovascular glaucoma
OA: Ophthalmic artery
OAO: Ophthalmic artery occlusion
OCT: Optical coherence tomography
OIS: Ocular ischemic syndrome
OVA: Ocular vascular accident
PB: Phthisis bulbi
PCA: Posterior ciliary artery
PDR: Proliferative diabetic retinopathy
PLLA: Poly-L-lactic acid
RAO: Retinal artery occlusion
VH: Vitreous hemorrhage
